# Supplementary material for: A Phase 1, Randomized, Double-Blinded, Placebo-Controlled and Dose-Escalation Study to Evaluate the Safety and Immunogenicity of the Intranasal DelNS1-nCoV-RBD LAIV for COVID-19 in Healthy Adults
Source: Vaccines (Basel). 2023 Mar 24;11(4):723. doi: 10.3390/vaccines11040723 (PMC10143096; doi:10.3390/vaccines11040723)
Supplement: Supplementary file 1 [file vaccines-11-00723-s001.zip › vaccines-2228525-supplementary.pdf]

vaccines

MDPI

Supplementary File: Laboratory Assays.

Methods

Treatment of saliva for enzyme immunoassay

Saliva specimens were collected using Salivette® (SARSTEDT, Nümbrecht, Germany). Saliva specimens were centrifuged at 1000 × g for 2 min, and then stored at −80°C until testing. On the day of testing, the saliva specimen was thawed and then centrifuged at 8500 rpm for 4 min. Saliva supernatant was treated with 1% Triton X-100 at room temperature for 30 min and diluted 1:1 with phosphate buffered saline (PBS).

Enzyme immunoassay for the detection of saliva total immunoglobulin

Enzyme immunoassay for the detection of saliva immunoglobulin was performed as described previously with modifications [1]. 96-well Nunc MaxisorpTM plates (Thermo Fisher Scientific; Cat#4424-04) were coated with either 200 ng/well (used for saliva preabsorption) or 100 ng/well (used for enzyme immunoassay). PierceTM avidin (Thermo Fisher Scientfic; Cat#21121) in 50 µL 0.05 M carbonate-bicarbonate buffer at 4°C for 16 hours and then blocked with blocking agent at 37°C for 2 hours. 100 ng biotinylated recombinant SARS-CoV-2 RBD (ABclonal; Cat#RP02326) in 50 µL 1× PBS were added to the wells with 200 ng avidin/well. For control wells, PBS was added instead biotinylated recombinant SARS CoV-2 RBD. The plates were incubated at room temperature for 30 min with shaking followed by washing. 50 µL-treated saliva was added to the wells with or without biotinylated RBD. For each plate, saliva specimen from an individual who has received BNT162b2 vaccine was added as a control. Antibody binding was performed for one hour with shaking at room temperature. After washing, 50 µL/well horseradish peroxidase conjugated goat anti-human IgG, IgM and IgA (Thermo Fisher Scientific; Cat#A18847) at a dilution of 1:2000 was added and incubated at room temperature for 30 min with shaking. 100 µL 3,3′,5,5′-tetramethylbenzidine single solution (Thermo Fisher Scientific; Cat#002023) was added to each well for signal development at dark for 10 minutes which was then stopped with 100 µL 0.3M sulfuric acid. The optical density (OD) was read at 450 and 620 nm.

Reference

1 Isho, B.; Abe, K.T.; Zuo, M.; Jamal, A.J.; Rathod, B.; Wang, J.H.; Li, Z.; Chao, G.; Rojas, O.L.; Bang, Y.M.; et al. Persistence of serum and saliva antibody responses to SARS-CoV-2 spike antigens in COVID-19 patients. *Sci. Immunol.* **2020**, *5*, eabe5511. https://doi.org/10.1126/sciimmunol.abe5511.
